# Cefepime-Induced Neuropsychiatric Manifestations in a Patient With Chronic Kidney Disease: A Case Report

**DOI:** 10.7759/cureus.11395

**Published:** 2020-11-09

**Authors:** Sai S Achi, Ian C White-Gittens, Anya Weerasinghe

**Affiliations:** 1 Internal Medicine, Harlem Hospital Center, New York, USA

**Keywords:** cefepime, neuropsychiatry symptoms, chronic kidney disease

## Abstract

Cefepime is a fourth-generation cephalosporin that has multitude of uses in various conditions. Cefepime associated neurotoxicity was first described in 1999. Here, we describe a case in which we appreciated the neuropsychiatric effects of cefepime on a patient who had underlying chronic kidney disease.

## Introduction

Cefepime is a fourth-generation cephalosporin antibiotic that provides coverage against Pseudomonas aeruginosa, other AmC beta-lactamase producing bacteria, and infections with both gram-negative and gram-positive aerobes. Given the majority of excretion is via the kidneys-the medication is renally dosed. Some adverse effects have been noted to be nausea, rash, delirium, and seizures [1]. Many of the noted side effects of cefepime do not include some of the neuropsychiatric manifestations that may occur especially in patients with kidney disease. The pathophysiology of how cefepime causes neurotoxicity has been attributed to inhibiting the gamma-aminobutyric acid-A (GABA-A) receptors potentially inhibiting the release of GABA which would explain the manifestations of seizures, myoclonus, or even global encephalopathy that is seen [2]. We present a case of an elderly immunocompromised gentleman who had been started on Cefepime during the hospital course and developed neuropsychiatric symptoms that resolved after the medication was discontinued. 

## Case presentation

A 70-year-old man with a past medical history significant for hypertension, diabetes mellitus, chronic kidney disease stage 4, and prostate cancer stage 4 on outpatient chemotherapy at another facility presented to our emergency department with complaints of vomiting and nausea a day prior to admission. He had three episodes of nonbloody, non-bilious, and nonbloody diarrhea as well. The patient had one-day history of productive cough with whitish sputum and subjective fevers. Patient, though adherent with chemotherapy, had admitted that he was not able to take his other home medications. In the ED, patient had a temperature of 103.1degrees F, heart rate of 98 beats per minute, blood pressure of 102/55 mmHg, respiratory rate of 18 breaths per minute, glucose of 121 mg/dl. Physical exam was significant for conscious, alert and oriented to person, place, and time; pink and moist mucosa, right surgical scar at the right hypochondriac, soft, nontender, bowel sounds were present. Pertinent abnormal labs include: white blood cell count (WBC), 14.5 units/mcl (reference range: 4.80-10.80 units/mcl) with neutrophils of 82.1% (reference range: 44%-70%), blood urea nitrogen (BUN) 39 mg/dl (reference range: 7-18 mg/dl), creatinine (Cr) 3.8 mg/dl (reference range: 0.7-1.2 mg/dl), calcium (Ca) 7.7 mg/dl (reference range: 8.5-10.1 mg/dl). Chest x-ray showed a focal area of consolidation involving the left midlung. Patient was admitted and managed on the medical floor for sepsis likely secondary hospital acquired pneumonia especially given his immunocompromised state. Patient was started on IV vancomycin and piperacillin-tazobactam in the emergency department.

During the course of the hospitalization, infectious disease was consulted and they recommended to start the patient on IV cefepime 1 g Q24 hours, oral linezolid 600 mg Q12H, and oral clindamycin 450 mg Q8H. In addition, patient was started on calcium acetate for his hypocalcemia and was continued on sodium bicarbonate 1300 mg Q8H for his AKI on CKD. After two days the cefepime dose was increased to 2 mg. Two days after increasing the dose of cefepime, patient started developing altered mental status. Patient was alert and oriented only to person, incoherent and expressed thoughts like “I would kill myself if I had the chance” so the cefepime was discontinued and within one day the patient was more conversant, denied any suicidal ideations and was alert and oriented to person and place. In addition, the BUN and creatinine (Cr) levels trended up to 41 mg/dl and 5.4 mg/dl. So the calcium acetate was switched to calcium carbonate and calcitriol. After making these changes the Cr level trended down to 3.4 mg/dl. In addition, the corrected Calcium level was at normal levels based on the albumin level of the patient. The mental status improved and patient was subsequently discharged. Table 1 provides the electrolyte levels.

**Table 1:** Lab values showing the electrolytes On August 2nd, the cefepime was increased to 2 gm; on August 10th, the cefepime was discontinued. Albumin values are also provided for corrected calcium levels.

	Reference Range and Units	2-Aug	10-Aug	12-Aug	13-Aug	14-Aug	17-Aug
Sodium (mmol/L)	136-145 mmol/L	144	151	151	147	146	140
Potassium (mmol/L)	3.5-5.1 mmol/L	4.09	4.28	4.82	4.7	4.4	4.6
Chloride (mmol/L)	98-107 mmol/L	116	115	117	109	109	105
Bicarbonate (mmol/L)	22-29 mmol/L	12	20	20	24	22	21
Glucose (mg/dL)	74-109 mg/dL	123	149	119	133	109	96
Blood Urea Nitrogen (mg/dL)	7-18 mg/dL	35	32	31	28	27	28
Creatinine (mg/dL)	0.7-1.2 mg/dL	3.9	4.9	4.5	4	3.7	3.5
Calcium (mg/dL)	8.5-10.1 mg/dL	7.1	6.4	6.5	6.6	6.7	6.5
Anion Gap (mEq/L)	6-18 mEq/L	16	16	15	14	15	14
Total protein (g/dl)	6.4-8.3 g/dL	7		8			8
Total bilirubin (mg/dl)	<=1.2 mg/dL	0.7		0.8			0.6
Alanine Aminotransferase (U/L)	<=33 U/L	20		21			20
Alkaline Phosphate (U/L)	35-104 U/L	70		68			72
Aspartate Aminotransferase (U/L)	<=32 U/L	16		18			16
Albumin (g/dl)	3.97-4.94 g/dL	3.3		3.4			3.5
Direct bilirubin (mg/dl)	0.0-0.3 mg/dL	0.3		0.5			0.4

## Discussion

Cefepime is a fourth-generation cephalosporin. Cefepime associated neurotoxicity was first described in 1999. Studies have indicated that up to 15% of patients in the intensive care unit (ICU) will experience the neurotoxicities [3]. Cefepime neurotoxicity is relatively common and often underdiagnosed and has been seen mainly in the Intensive Care Unit setting with symptoms occurring four days after initiation of treatment and most common symptoms include decreased consciousness levels, myoclonus, and confusion [4]. In addition, as cefepime is renally excreted, studies have shown that a reduced renal function will reduce the clearance of cefepime from the body [5]. In 2012, the Food and Drug Administration (FDA) had recommended that the cefepime dose should be adjusted for patients with renal disease as there is an association with status epilepticus. This recommendation had resulted from the FDA reviewing the database from the years 1996 to 2012 with 59 reported cases of nonconvulsive epilepticus cases during the administration of cefepime; of those patients who received cefepime, 58 patients had renal impairment of which 56 patients didn’t have renally dosed cefepime. Symptomology of the patients mainly were decreased level of consciousness, myoclonus, confusion, aphasia, seizure, and agitation. The treatments that were adopted were discontinuing the antibiotic, trial of benzodiazepines which opposed the cefepime’s antiGABA mechanism, or hemodialysis session which cleared cefepime’s levels up to 70% [6,7]. A study was conducted and indicated that eight patients with renal failure given cefepime developed neurotoxicity that ranged from decreased level of consciousness to seizures; of the eight patients, three indicated clinical improvement after cefepime was discontinued [8]. Another study shows that the neurological effects and the notion of hallucinations are reversible once the offending agent is taken out which was seen in our patient as well [9].

However, in our patient, his symptoms were atypical. As previously mentioned Cefepime’s neurotoxicity was associated with anti-GABA receptor activity, while our patient’s symptoms appeared to be more Dopaminergic in nature given his psychotic features. Previously psychotic features have only been described in patients using Quinolones and Trimethoprim-Sulphamethoxazole. 

Some limitations that exist in our case report does include confounders do exist that could also have contributed to the neuropsychiatric changes in our patient such as delirium/confusion in hospitalized elderly patients. A study expresses that manifestations of delirium can range from incoherence to poor cognition or even hallucinations [10]. Other limitations do include as well electrolyte abnormalities such as the hypernatremia that was seen for a couple of days during the time that the cefepime was also discontinued; studies mention some of the side effects of dysnatremia include drowsiness/confusion or lethargy [11]. Thus the resolution of symptoms could also be accounted for correcting the electrolyte disorders as well.

This case report urges the clinician to consider that in patient who is on cefepime with sudden progressive deterioration in mentation that there is a possibility of the antibiotic contributing to the neurotoxicity especially in patients with kidney disease as well in the differential diagnoses. It is also imperative that patients on cefepime in an ICU setting that are Confusion Assessment Method (CAM) positive should have the dose adjusted or medication discontinued with observation for improvement as we believe it may be a frequently underdiagnosed condition [12].

## Conclusions

Given this interesting case and the literature about cefepime and neurotoxicity, the neuropsychiatric side effects should be considered as potential side effects whenever administering the medications in patient who had kidney disease. In addition, a focused neurological exam and mental status exam as well in patients who are on cefepime in order to identify possible side effects of the medication and make appropriate changes to the antibiotic regiment if a patient is to develop side effects from cefepime.
